# Effects of inhaled nitric oxide on hemostasis in healthy adults treated with heparin: a randomized, controlled, blinded crossover study

**DOI:** 10.1186/1477-9560-10-1

**Published:** 2012-01-09

**Authors:** Brahm Goldstein, James Baldassarre, Joseph N Young

**Affiliations:** 1 Research and Development, Ikaria, Inc., 6 Route 173, Clinton, NJ 08809 USA

**Keywords:** bleeding time, hemostasis, heparin, methemoglobin, nitric oxide, platelet aggregation

## Abstract

**Background:**

Effects of nitric oxide (NO) on hemostasis have been studied in various investigational settings, but data regarding inhaled NO on bleeding and platelet function are conflicting. It is not known if inhaled NO has an effect when administered with drugs that influence hemostasis. This trial evaluated effects of inhaled NO on hemostasis in the presence of heparin using aspirin as a positive control.

**Patients/Methods:**

Twelve healthy adult males were enrolled in a single-center, randomized, single-blind, four-way crossover trial. Subjects received 80 ppm NO or medical air (placebo) inhalation for 30 min with simultaneous injection of placebo or heparin. Aspirin capsules were used as a positive control. Parameters of hemostasis were measured before treatment and at post-treatment intervals.

**Results:**

Activated clotting time (ACT), prothrombin time (PT) and activated partial thromboplastin time (aPTT) increased only in groups that received heparin. Areas under the curve for ACT in heparin groups receiving inhaled NO were judged to be equivalent to those receiving medical air for both 0- to 4-h (ratio: 1.00; 90% CI, 0.90-1.11) and 0- to 24-h time intervals (ratio: 1.01; 90% CI, 0.92-1.12). Changes in bleeding time and platelet aggregation were observed only in aspirin groups. No clinically significant changes in hemoglobin, red blood cell counts or haematocrit were observed in any group.

**Conclusions:**

Inhaled NO, when administered with heparin, exhibited no significant additive effects on ACT, PT, aPTT, bleeding time or platelet aggregation.

## Introduction

Nitric oxide (NO) is produced endogenously and plays an important role in a range of physiologic functions including the regulation of vascular smooth muscle tone, as well as the modulation of platelet function via the guanylate cyclase signaling pathway [1]. Inhaled NO is approved for the treatment of term and near-term (> 34 weeks) neonates with hypoxic respiratory failure associated with clinical or echocardiographic evidence of pulmonary hypertension, where it selectively relaxes the pulmonary vasculature, reduces right to left shunting of poorly oxygenated blood, and thereby improves oxygenation [2-4]. The vasodilatory effects of inhaled NO are localized to the pulmonary vasculature because NO is immediately converted to nitrite and methemoglobin [5]. However, there are potential but as yet hypothetical systemic effects of inhaled NO, including possible peripheral effects on platelet function and bleeding, that have prompted a variety of studies.

Early studies in healthy human volunteers and animals demonstrated prolonged bleeding times following inhalation of NO [6,7]. While some later studies were confirmatory of these findings [8-10], others found no effect on bleeding time [11,12] or found an increase in bleeding time only after prolonged (e.g. 55 min) exposure to inhaled NO [13]. Studies of the effects of inhaled NO on bleeding in adults and neonates with respiratory complications have been similarly conflicting. Some investigators reported increased bleeding time [14,15], whereas others reported no increase in bleeding time or the incidence of clinically significant hemorrhage [16-18].

With regard to platelet aggregation, the effects of inhaled NO have been investigated in animals, healthy adults, and neonates. Again, this diverse group of studies has yielded conflicting results. Some investigators have reported inhibition of platelet function or aggregation [10,14,18-24], while others have found little or no effect [8,11-13,15,25].

While studies examining the effects of inhaled NO on bleeding and platelet function have yielded inconsistent results, it is also not known if inhaled NO has an additive or synergistic effect when given in combination with other drugs that influence hemostasis. The clinical implications of the potential adverse effects of inhaled NO on hemostasis may be significant in surgical settings where anticoagulants may be employed and in neonates who are placed on anticoagulation therapy for extracorporeal membrane oxygenation. In both circumstances, patients may have received inhaled NO before, during, or after anticoagulation therapy. To address these issues, this study was designed to evaluate effects of inhaled NO on coagulation and platelet aggregation in the presence and absence of heparin, with aspirin as a positive control.

## Methods

### Patients and study design

This was a single-center, randomized, single-blind, four-way crossover study conducted at Inveresk Clinical Research (ICR), Edinburgh, Scotland. The study was approved by the Independent Ethics Review Committee of Inveresk Research and all subjects gave written informed consent. The study was conducted in accordance with Guidance on Good Clinical Research Practice in the European Community and the International Conference on Harmonisation of Technical Requirements for Registration of Pharmaceuticals for Human Use. Quality control and quality assurance were carried out by ICR.

The study was initiated in November 1998 and was completed in December 1998. Healthy, non-smoking males aged 18 to 50 years were considered eligible for inclusion. The use of any investigational drugs (≤4 months prior to study entry), the need for prescription or over-the-counter drugs (≤7 days prior to study entry), a history or suspicion of bleeding disorders, recent surgery or significant trauma (≤3 months prior to screening) and blood donation or loss (> 400 ml; ≤12 weeks prior to study entry) were among the exclusion criteria (Table 1).

**Table 1:** Study Exclusion Criteria

Exclusion Criteria
Administration of any investigational drug in the period 0 to 3 months before entry to the study (0 to 4 months if the previous investigational drug was a new chemical entity)

A need for any medication (including OTC drugs, e.g., aspirin) during the period 0 to 7 days before entry to the study

Existence of any surgical or medical condition which, in the judgment of the clinical investigator, might interfere with the absorption, distribution, metabolism or excretion of the drug

Presence or history of allergy requiring treatment

Donation or loss of greater than 400 ml of blood in the period 0 to 12 weeks before entry to the study

Serious adverse reaction or hypersensitivity to any drug, especially heparin

History of easy bruising

Personal or family history or coagulation or bleeding disorders, cerebral hemorrhage, or reasonable suspicion or vascular malformations, including aneurysms

History of important bleeding, e.g., hematemesis or melena, severe or recurrent epistaxis or hemoptysis

Known heparin resistance

Consumption of aspirin in any form, or of other non-steroidal anti-inflammatory drugs within the 3 weeks prior to the final eligibility check

Rectal bleeding, including that presumed from hemorrhoids within 3 months prior to screening

Surgery or significant trauma within the 3 months prior to screening

Significant epigastric pain or indigestion either chronically or within 4 weeks prior to screening

Platelet aggregation response of 40% to 10 μmol/L ADP and 2 μg/mL collagen

Abnormal platelet aggregation response to arachidonic acid

Inability to communicate or co-operate with the investigator because of a language problem, poor mental development or impaired cerebral function

Smokers

Objection by the subject's general practitioner to his/her patient's participation in the study

Four treatment combinations were employed as follows:

1. Heparin injection + placebo inhalation + placebo capsules (HPP).

2. Placebo injection + NO inhalation + placebo capsules (PNP).

3. Heparin injection + NO inhalation + placebo capsules (HNP).

4. Placebo injection + placebo inhalation + aspirin capsules (PPA).

The study was conducted in four successive treatment periods, with patients randomly assigned to receive a different treatment combination during each treatment period. Randomization was performed via computer-generated, 4-digit sequences, corresponding to the treatment combinations listed above. Randomization codes were held by the study pharmacist, who was the sole preparer of dosing and kept randomization blinded to all members of the study team.

There was a 7- to14-day washout period between treatments. The primary end point was activated clotting time (ACT), determined via area under the curve (AUC) for the HPP and HNP treatment combinations over a period of 24 h (AUC_0→24_). This measure was chosen as the primary end point based on its use in monitoring heparin anticoagulation at the time of study; given that the intent of the study was to provide insight as to the impact of concomitant use of inhaled NO and heparin on hemostasis, the choice of ACT was deemed to be appropriate.

Secondary analyses included AUC measurements of ACT for the PNP and PPA combinations over the first four hours (AUC_0→4_), as well as pharmacodynamic parameters that included activated partial thromboplastin time (aPTT), prothrombin time (PTT), bleeding time, creatinine clearance, and methemoglobin levels.

Injections were given as 5 ml of either heparin (5,000 i.u.) or 0.9% sodium chloride (placebo), administered as an intravenous bolus injection. Capsules were administered orally as 2 × 300 mg aspirin or matching placebo (lactose) with 200 ml of water, 15 min prior to the simultaneous administration of injections and inhalations. The doses of both heparin and aspirin were based on recommended doses to be used in clinical situations at the time of the study. Gas inhalations were either 80 ppm NO or medical air (placebo) administered for 30 ± 1 min by an lNOvent^® ^delivery system (Datex-Ohmeda, Inc.; Madison, WI) with an inspired air flow of 8 ± 2 L/min. The doses of inhaled NO and aspirin were based on previous studies of this type in healthy volunteers [11,13].

### Nitrogen-based Gas Measurements

Nitric oxide and nitrogen dioxide (NO_2_) were measured by the INOvent^® ^delivery system at 5, 10, 15, and 30 min after commencement of gas delivery.

### Laboratory tests

Serial blood samples were collected via cannula placed in subjects' forearms, which were flushed with saline; approximately 175 mL of blood was obtained from each subject over the duration of the study. Samples for clinical chemistry and hematology were collected in heparinized tubes (5.0 ml), EDTA-coated tubes (3.0 ml) and in citrated anticoagulant tubes (2.7 ml). Hemoglobin (Hb), total red blood cell (RBC) count and hematocrit (Hct) were performed with a Technicon H1 Analyser (Bayer/Technicon Instruments; Tarrytown, NY). Tests were performed immediately before and 24 h after commencement of each treatment period, and 7 days after the final treatment period.

### ACT, aPTT, PT, and Heparin Concentration

Standard analyses for ACT (Hattersley method [26]; kaolin activator), aPTT (Larrieu and Weiland method [27]), and PT (Biggs and Macfarlane method [28]) assays were performed immediately before dosing and at intervals following commencement of gas inhalation (5 min, 10 min, 15 min, 30 min, 1 h, 2 h, 4 h, 8 h [excluding ACT], and 24 h [± 2 h]).

Heparin concentration was determined based on anti-factor Xa activity using a chromogenic substrate assay.

### Bleeding Times

Bleeding times were measured using the modified Ivy Nelson method immediately prior to dosing and at 10 min, 30 min, 1 h, 2 h, 4 h, and 12 h after commencement of gas inhalation.

### Platelet Aggregation

Platelet aggregation was induced by collagen (2 μg/ml) or adenosine diphosphate (ADP) (10 μM), and measurements were executed by Born light aggregometry. Agonist-induced aggregation was quantified via measurement of maximal light transmission (measured as a percentage). Venous blood samples (10 ml) were taken immediately prior to dosing and 30 min, 1 h and 24 h following commencement of gas inhalation.

### Methemoglobin

Methemoglobin concentrations in venous blood were measured immediately prior to dosing and 30 min, 1 h and 2 h following commencement of gas inhalation with an IL-682™ CO-oximeter (Instrumentation Laboratory, Brussels, Belgium).

### Cyclic GMP

Plasma cyclic guanosine monophosphate (cGMP) levels were measured in samples taken at -5, -10, +5, +45 and +60 min relative to commencement of inhalation. Blood was collected into chilled heparinized tubes, placed on ice, then centrifuged at 2500 rpm for 5 min. Plasma was then separated, with 1.5 mL placed in a polypropylene tube and the remainder in a second tube. Samples were stored at -70°C and then assayed at the Department of Clinical Pharmacology at Lund University in Sweden. Estimations for cGMP were assayed in a subgroup of 6 subjects (during Treatment Period 3 only) as a marker of biological activity and were not considered pivotal study data.

### Statistical analysis and sample size determination

Summary statistics (i.e. arithmetic mean, median, standard deviation, minimum, maximum and n) were calculated for all treatment combinations and all measurements. The primary analysis end point was defined to be the activated clotting time (ACT). A curve representing ACT versus time was produced, and AUC _0→4 _and AUC _0→24 _was calculated for each subject and dosing session. Following logarithmic transformation, the AUC values were subjected to analysis of variance, including terms for subject, period and treatment combination. For the primary analysis, a point estimate and 90% confidence interval for the difference between the HNP and HPP combinations were constructed. If the 90% confidence interval for the measure of relative "bioavailability" (i.e. AUC ratio of the HNP combination relative to the HPP combination) lay within the acceptance range of 0.80 to 1.25, the two combinations of interest were judged equivalent. For the secondary end point, HNP was also compared with PNP and PPA using similar methods.

### Sample size determination

In a previous, unpublished study in healthy volunteers (Data on file; Ikaria, Inc., Clinton, NJ), the mean ACT value after heparinization was 269 s, with an associated between-subject standard deviation of 27.5 s. Assuming a correlation coefficient of 0 between ACT values from two treatment periods in the same subject, the standard deviation associated with the difference is 38.9 s. A total of seven subjects was required in order to have a reasonable chance (i.e. power 90%) that the 90% confidence interval of the measure of relative bioavailability (i.e. AUC ratio) would lie within the acceptance range of 0.80 to 1.25, assuming that the two combinations of interest are equivalent. Based on considerations of differences between the previous study and this study, a sample size of 12 was recommended.

## Results

### Patients and Demographics

Twelve subjects entered the study, with one subject withdrawing after receiving the HPP combination in the first treatment session. All subjects were healthy white males with a mean age of 33.6 years ± 11.9 SD, mean height of 179.1 cm ± 6.2 SD, and mean weight of 80.2 kg ± 11.7 SD (Table 2).

**Table 2:** Patient Demographics

Patient*	Treatment Sequence	Age	Height (cm)	Weight (kg)
001	HPP/PNP/PPA/HNP	34	181	77.9

002	HNP/PPA/PNP/HPP	28	170	74.4

003	PNP/HNP/HPP/PPA	46	181	93.9

004	PPA/HPP/HNP/PNP	23	179	64.7

005	HPP/PNP/PPA/HNP	49	179	84.3

006	PNP/HNP/HPP/PPA	47	176	82.5

007	PPA/HPP/HNP/PNP	26	181	88.4

008	HNP/PPA/PNP/HPP	41	193	104.3

009	HNP/PPA/PNP/HPP	48	175	70.9

010	HPP/PNP/PPA/HNP	20	180	69.7

011	PNP/HNP/HPP/PPA	19	170	67.7

012	PPA/HPP/HNP/PNP	22	184	84.1

*All patients were white males.

HNP, heparin injection + nitric oxide inhalation + placebo capsules; HPP, heparin injection + placebo inhalation + placebo capsules; PNP, placebo injection + nitric oxide inhalation + placebo capsules; PPA, placebo injection + placebo inhalation + aspirin capsules.

### Primary end point - ACT

**Figure 1:**
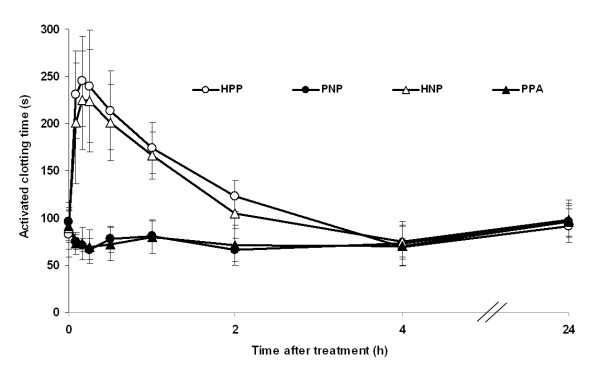
**ACT before and at various time points after treatment with inhaled nitric oxide or placebo gas**. Data points represent mean ± SD. ACT, activated clotting time; HNP, heparin injection + nitric oxide inhalation + placebo capsules; HPP, heparin injection + placebo inhalation + placebo capsules; PNP, placebo injection + nitric oxide inhalation + placebo capsules; PPA, placebo injection + placebo inhalation + aspirin capsules.

Over the first 4 h, ACT was higher in groups that received heparin injection (HPP and HNP groups) than in groups that received placebo injection (PNP or PPA), but all four treatment combinations appeared similar from 4 to 24 h post-treatment (Figure 1). AUC analysis was performed for ACT vs. time for data collected from 0-4 h post-treatment and for 0-24 h post-treatment. Results are summarized in Table 3. For the 0- to 4-h interval, the ratio of the AUCs of the HNP combination relative to the HPP combination was 1.00 with a 90% confidence interval of 0.90 to 1.11, within the acceptance range of 0.80 to 1.25 (Table 3). Consequently, the two combinations were judged equivalent, and the addition of inhaled NO did not change ACT when given together with a heparin injection. The HNP and HPP combinations were also judged to be equivalent for the 0-24 h interval. The AUC values for the HNP combination were, on average, 76% higher than the PNP combination for the 0- to 4-h interval and indicated that the addition of heparin increased ACT (Table 3). Similarly, the AUC values for the HNP combination over the first 4 h were, on average, 79% higher than the PPA combination, indicating that adding a heparin injection increased ACT as compared with aspirin (Table 3). However, these differences were less for the 0- to 24-h interval.

**Table 3:** Area Under the Curve for Activated Clotting Time

	0-4 h		0-24 h	
	Means*	**Comparison**	Means*	**Comparison**

**Treatment**	**(s·h)**	**Ratio^† ^(90% CI)**	**(s·h)**	**Ratio^† ^(90% CI)**

HPP	526.75	1.00 (0.90, 1.11)	2,181.53	1.01 (0.92, 1.12)

PNP	297.70	1.76 (1.58, 1.96)	1,934.88	1.14 (1.03, 1.26)

HNP	524.43	-	2,206.74	-

PPA	292.55	1.79 (1.61, 1.99)	1,922.91	1.15 (1.04, 1.27)

CI, confidence interval; HPP, heparin injection + placebo inhalation + placebo capsules; HNP, heparin injection + NO inhalation + placebo capsules; NO, nitric oxide; PNP, placebo injection + NO inhalation + placebo capsules; PPA, placebo injection + placebo inhalation + aspirin capsules.

*Geometric means from analysis of variance.

^†^Ratio defined as HNP divided by relevant other combination (after exponential transformation of difference on logarithmic scale).

### Adverse events and safety

No serious adverse events (AEs) were reported. The only AE reported was that of vomiting, which occurred in a patient who received the PNP combination.

### PT and aPTT

**Figure 2:**
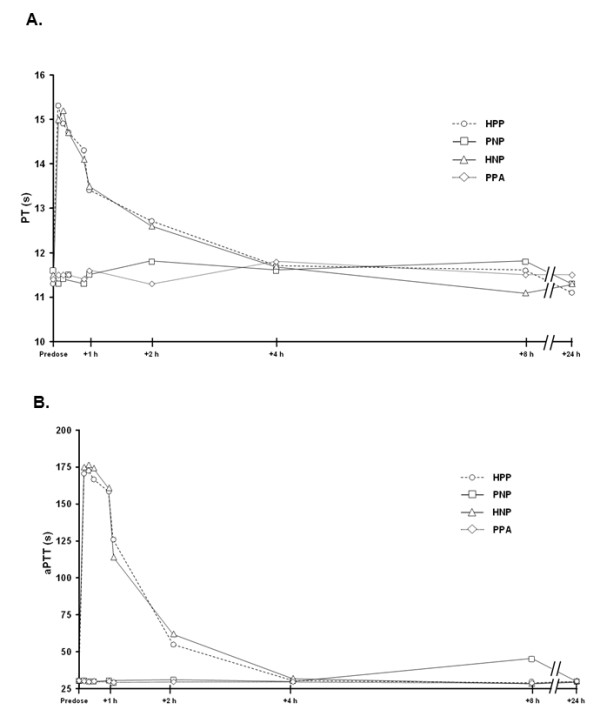
**(A) PT and (B) aPTT before and at various time points after treatment with inhaled nitric oxide or controls**. Data points represent mean ± SD. aPTT, activated partial thromboplastin time; HNP, heparin injection + nitric oxide inhalation + placebo capsules; HPP, heparin injection + placebo inhalation + placebo capsules; PNP, placebo injection + nitric oxide inhalation + placebo capsules; PPA, placebo injection + placebo inhalation + aspirin capsules; PT, prothrombin time.

The mean results for PT and aPTT showed an increase after injection only in the groups that received heparin (HPP and HNP), with values returning to near baseline by 4 h (Figure 2).

### Heparin Concentration

**Figure 3:**
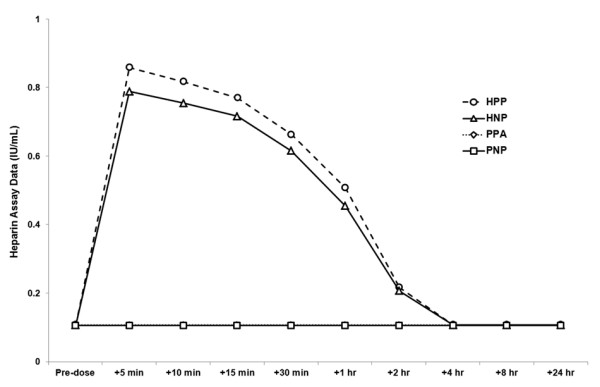
**Heparin assay data/anti-Xa activity before and at various time points after treatment with inhaled nitric oxide or placebo gas**. Data points represent mean. HNP, heparin injection + nitric oxide inhalation + placebo capsules; HPP, heparin injection + placebo inhalation + placebo capsules; PNP, placebo injection + nitric oxide inhalation + placebo capsules; PPA, placebo injection + placebo inhalation + aspirin capsules.

Increased anti-factor Xa activity was measured from 5 min to 2 h post-treatment in the groups that received heparin injection (Figure 3), and there was no evidence of anti-factor Xa activity in the groups that received placebo injection. In addition, there was no evidence of additional potentiation of anticoagulant effects when heparin and NO were administered simultaneously.

### Bleeding Time

**Figure 4:**
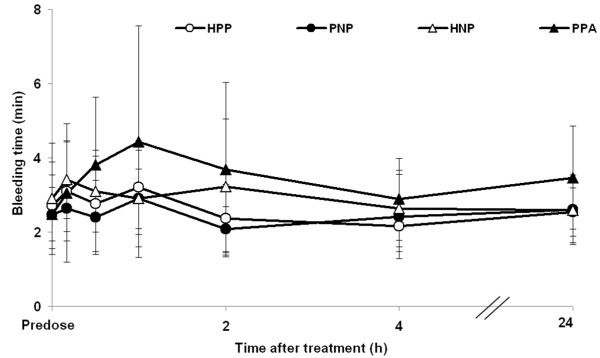
**Bleeding time before and at various time points after treatment with inhaled nitric oxide or placebo gas**. Data points represent mean ± SD. HNP, heparin injection + nitric oxide inhalation + placebo capsules; HPP, heparin injection + placebo inhalation + placebo capsules; PNP, placebo injection + nitric oxide inhalation + placebo capsules; PPA, placebo injection + placebo inhalation + aspirin capsules.

Extended bleeding times were noted from 10 min to 2 h post-dose for the group treated with aspirin (PPA group) (Figure 4), but not in any of the other groups. In general, changes were mild, and few values fell outside normal limits. Data from the other treatment groups (i.e. HPP, PNP and HNP) were within expected limits throughout the study.

### Platelet Aggregation

**Figure 5:**
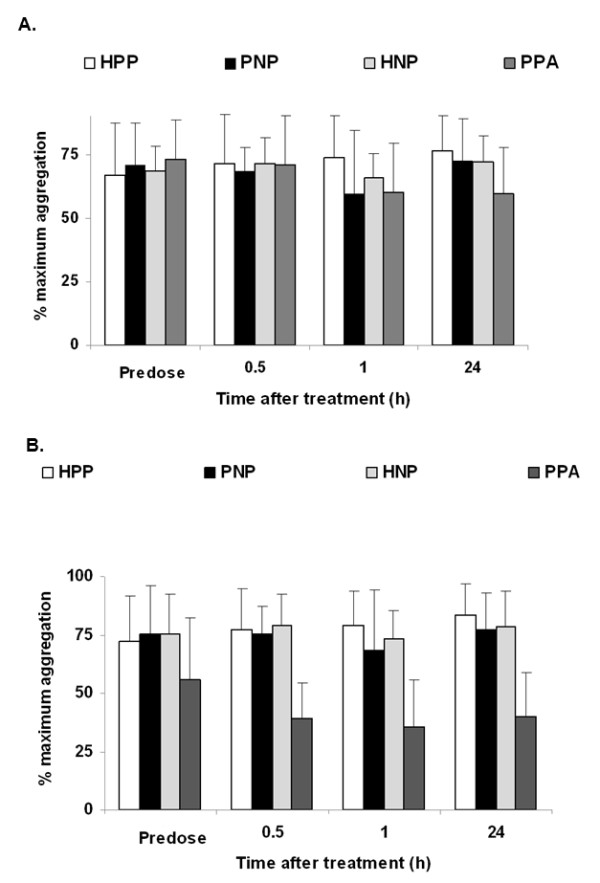
**Platelet aggregation before and at various time points after treatment with inhaled nitric oxide or placebo gas**. Data points represent mean + SD. ADP, adenosine diphosphate; HNP, heparin injection + nitric oxide inhalation + placebo capsules; HPP, heparin injection + placebo inhalation + placebo capsules; PNP, placebo injection + nitric oxide inhalation + placebo capsules; PPA, placebo injection + placebo inhalation + aspirin capsules.

Effects on platelet aggregation were restricted to the group treated with aspirin (PPA) (Figure 5). Response to ADP was generally unaffected, with no changes in platelet aggregation in the HPP, PNP, and HNP groups (Figure 5A). Individual responses to a single dose were variable, but several subjects experienced inhibition of collagen-induced platelet aggregation (Figure 5B), typical for the pharmacologic action of aspirin.

### Cyclic GMP

**Figure 6:**
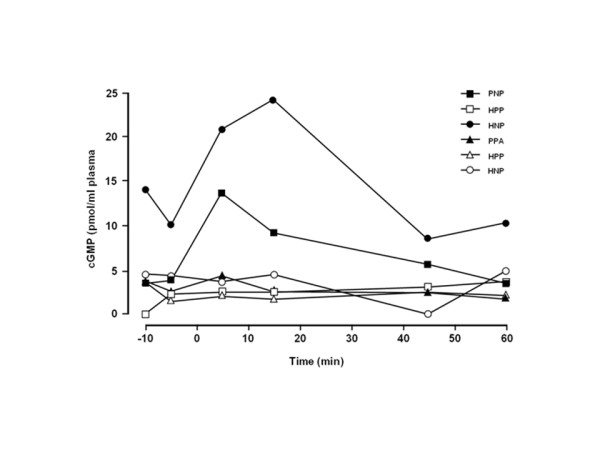
**cGMP levels before and at various time points after treatment**. HNP, heparin injection + nitric oxide inhalation + placebo capsules; HPP, heparin injection + placebo inhalation + placebo capsules; PNP, placebo injection + nitric oxide inhalation + placebo capsules; PPA, placebo injection + placebo inhalation + aspirin capsules.

As mentioned previously, 6 patients had cGMP estimations conducted during Treatment Period 3 (Figure 6); these measurements were performed as a marker of biological activity and were considered non-pivotal data. Among the 6 patients, 4 had cGMP levels that remained relatively stable. Two patients (PNP and HNP) had cGMP elevations that peaked at 5 and 15 min, respectively, and then returned to near-baseline levels by the end of the treatment period (min 45-60).

### Hematology

Hemoglobin, RBC and Hct levels were assessed immediately prior to study dose and 24 h post-dose. No clinically significant changes from baseline were found for mean values for any of the study combinations. In some subjects, there was a decrease in Hb and Hct as the study progressed, but this was presumed to be due to repeated venesection.

### Creatinine

Increases from baseline mean plasma creatinine were observed in the HPP (+13.0 ± 14.3 μmol/L), PNP (+15.9 ± 12.3), HNP (+17.0 ± 14.0), and PPA (+8.9 ± 12.5) groups at hour 12. By hour 24, no substantial changes from baseline in creatinine levels were noted (+4.0 ± 4.7, +7.2 ± 5.7, +3.8 ± 5.4 and +4.9 ± 5.3, respectively). Nine patients (2 in the HPP, HNP and PPA groups and 3 patients in the PNP group) exhibited above-normal creatinine levels (upper limit of normal: 122 μmol/L) at hour 12. Two patients (one in HPP and one in PNP) had above-normal creatinine levels at 24 h.

### Methemoglobin

**Figure 7:**
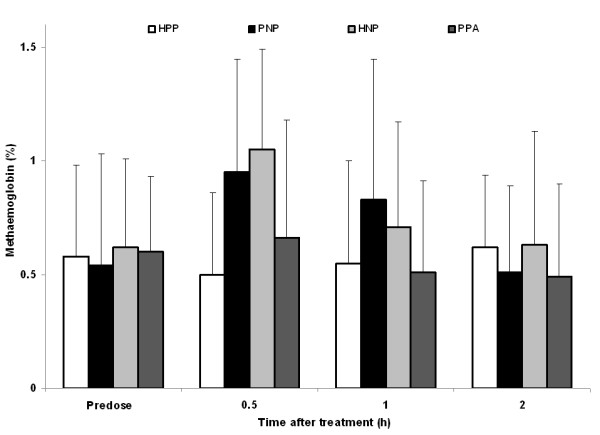
**Methemoglobin levels before and at various time points after treatment with inhaled nitric oxide or placebo gas**. Data points represent mean + SD. HNP, heparin injection + nitric oxide inhalation + placebo capsules; HPP, heparin injection + placebo inhalation + placebo capsules; PNP, placebo injection + nitric oxide inhalation + placebo capsules; PPA, placebo injection + placebo inhalation + aspirin capsules.

Mean methemoglobin (metHb) values showed no significant changes when placebo inhalation was given (HPP, PPA) but in the PNP and HNP groups, the mean percentage increased to maximum values at +30 min (0.95% and 1.05%, respectively) and returned to baseline after 2 h (Figure 7). All metHb levels were within normal limits except for slight increases in some subjects. Only two subjects experienced Hb levels over 1.5%; one during all four combination post-dose measurements (maxima of 1.9% at 30 min post-PPA, 1.6% at 1 h post-HPP, 2.0% at 30 min post-HNP and 2.4% at 1 h post-PNP), with the second patient at 30 min post-HNP dose (1.6%).

## Discussion

This investigation of the effects of inhaled NO in healthy male volunteers found no significant additive effect on hemostasis, as measured by aPTT and bleeding time, when administered in combination with a standardized dose of heparin. Analysis of the primary end point of ACT revealed that the ratio of the AUCs of the groups that received HPP to those that received HNP indicated that the treatments were equivalent with regard to this parameter. Consequently, the addition of NO did not alter the effect of heparin on ACT. Bleeding time, PT, aPTT and platelet aggregation were investigated to further address the effects of inhaled NO on hemostasis. PT and aPTT were prolonged only in those groups receiving heparin; only groups receiving aspirin demonstrated prolonged bleeding times or inhibition of platelet aggregation. As a result, following investigation of the effects of inhaled NO on multiple measures of hemostasis, we found no notable alterations to hemostasis with the use of inhaled NO, including during combination use with heparin.

In this study, NO inhalation was tolerated well and resulted in no clinically significant changes from baseline in safety criteria including metHb levels. The only AE reported was that of vomiting in one patient while receiving PNP, deemed possibly related to study medication. Mean plasma creatinine levels increased over the first 12 h following study treatment but there was no sequence, period, or combination effect. This may indicate that the increase was probably a result of study participation but not study drug administration. The fasting period was different for each subject, and the creatinine results may be a reflection of dehydration or timing of sampling.

The subject population was that of healthy adult males; consequently, results may not be generalized to other patient groups, such as neonates, or in clinical settings such as surgery, where underlying developmental, pathophysiologic, or clinical conditions may be present. In addition, caution should be taken in extrapolating these results to women, although there is no current clinical evidence to suggest that gender-based differences exist in terms of NO effects on platelet function. A study by Gries et al examining the effects of inhaled NO on platelet function in 18 males and 18 females found no clear difference in effect between genders [9]; nevertheless, applicability to a female population is unknown and would be a valuable attribute to future study in this area.

The inhaled NO dose utilized for this study was 80 ppm, which is substantially greater than the typical recommended dose of 20 ppm. It should be noted, however, that in clinical practice, the dose range for inhaled NO is between 1 and 80 ppm As a result, the highest dose with in this range was chosen for this study to ensure the maximum potential effect of iNO in terms of the clinical efficacy and safety end points examined in this trial.

Exposure time to inhaled NO in this study was relatively short (30 min); this may be a limitation, as most clinical applications involve more prolonged exposure to inhaled NO. One can presume, however, that longer exposure times to inhaled NO would not have contributed any additional physiologic or safety-based effects in this study, particularly given the fact that inhaled NO has a very short half-life (seconds) and reaches steady-state within 5 to 10 min. In addition, inhaled NO is a selective pulmonary vasodilator and does not exhibit systemic effects. While inhaled NO has a duration of effect on the order of seconds to minutes, the effects of heparin are longer and dose-dependent--at the heparin doses administered in this study, duration of effect was expected to be approximately 60 min.

Standard laboratory tests that were utilized in similar trials published at the time of study were performed, including ACT, aPTT, PT, bleeding time, and anti-factor Xa activity measurements. These measurements are conventional standards in the context of measuring coagulation, but can be limited in terms of the impact of sampling technique (e.g., tube underfilling, sample contamination) and the use of platelet-poor plasma. Since the time of this study, new insights regarding coagulation have emerged, highlighting discrepancies between in vitro-based and in vivo-based processes regarding coagulation. While the standard tests provide a common, laboratory-based assessment of coagulation, the advent of more recent, near-patient/point-of-care focused testing systems have begun to comprise current clinical practice. While not preferred approaches in the clinical trial setting during the time of our study, tests such as Hemochron-ACT, thromboelastography, platelet function analysis, and portable INR/PT testing should be considered when conducting future trials in this area.

One of the key areas of interest when examining the literature on hemostasis-related effects of inhaled NO is the heterogeneity of the study designs, patient populations, controls, and measurements utilized in the various trials to date. Current medical literature with human subjects in this regard is limited in scope, with reports restricted to small patient trials (< 40 patients), case reports, and specific disease states [7-9,11,13,15,18-22]. Even when examining those trials conducted solely in healthy volunteers, the clinical conclusions appear contradictory. This study was specifically designed to begin to provide clarity on specific clinical scenarios by examining the effect of inhaled NO in a specific and common controlled, clinical setting.

Results from this study are in accord with two trials in which aspirin was employed as a control in healthy volunteers, where inhaled NO inhalation (30 ppm and 80 ppm for 15 to 30 min) was shown to have no influence on bleeding time or platelet function, while prolonged (55 min) exposure to 30 ppm resulted in slight, but significant (p = 0.04), increases in bleeding time [11,13]. In a third study, endogenous and inhaled NO did not influence the function of circulating platelets (i.e. P-selectin expression, platelet aggregability, and fibrinogen binding), most likely due to its rapid inactivation in the blood. Bleeding time was found to be increased significantly with NO inhalation; however, it was considered moderate in nature and of limited clinical relevance [8].

These results do not reproduce the results of three other trials in healthy volunteers [7,9,20], which demonstrated inhibition of platelet aggregation markers (as described above) with the use of inhaled NO, as well as prolonged bleeding time in the two trials where measured [9,20,29].

This study and the subsequent published research suggest that the effect of inhaled NO on bleeding time and other parameters of hemostasis, at clinically relevant doses, is modest at best, and of little clinical significance. Combined with the clinical literature that followed this study, these data may serve as a foundation for further experiments in a more clinical context (e.g., common clinical settings or patients with specific disease states), as was done in part with the heparin group in this study.

The wide-ranging experimental evidence surrounding the effects of inhaled NO on platelet aggregation, bleeding time, and other hemostasis-based parameters has raised vigilance in the monitoring of related potential AEs (e.g. hemorrhage) in a setting where inhaled NO is used. The clinical implications of this study in particular indicate that use of inhaled NO does not significantly impact parameters related to hemostasis such as aPTT, ACT, PT or bleeding time, even when administered in combination with heparin. This is consistent with the clinical experience that the use of inhaled NO in the setting of both adult and pediatric cardiovascular surgery, with or without anticoagulation, is not associated with clinical bleeding. Those patients are typically maximally anticoagulated with heparin both during and after surgery--a scenario similar to that which was tested in this study. Thus, the results of this pharmacodynamic study serve to provide additional evidence that inhaled NO does not predispose to clinical bleeding.

## Conclusions

Inhalation of 80 ppm inhaled NO for 30 min was well tolerated and was not associated with any additive effects on ACT when administered with heparin. In addition, other parameters related to hemostasis and platelet aggregation (e.g. bleeding time, PT, aPTT) were unaffected by inhaled NO.

## Conflict of interests disclosure

Brahm Goldstein, James Baldassarre, and Joseph N. Young are employees of Ikaria, Inc.

## Authors' contributions

BG, JB, and JNY contributed to the study conception and design, collected and assembled study data, analyzed and interpreted the data, contributed to the writing and revising of the manuscript, and provided final approval of the manuscript. JNY performed statistical analyses. All authors read and approved the final manuscript.
